# Supplementary material for: Deciphering the Proteome and Phosphoproteome of Peanut (Arachis hypogaea L.) Pegs Penetrating into the Soil
Source: Int J Mol Sci. 2025 Jan 14;26(2):634. doi: 10.3390/ijms26020634 (PMC11765555; doi:10.3390/ijms26020634)
Supplement: Supplementary file 1 [file ijms-26-00634-s001.zip › Supplementary Figures S2 and S3.pdf]

Supplementary Figure S2: the top 50 differential proteins with the closest interaction relationship were screened to map the protein interaction network.

Supplementary Figure S3: the DRPP interaction network database.
