# Supplementary figures and images for: Deciphering the Proteome and Phosphoproteome of Peanut (Arachis hypogaea L.) Pegs Penetrating into the Soil
Source: Int J Mol Sci. 2025 Jan 14;26(2):634. doi: 10.3390/ijms26020634 (PMC11765555; doi:10.3390/ijms26020634)

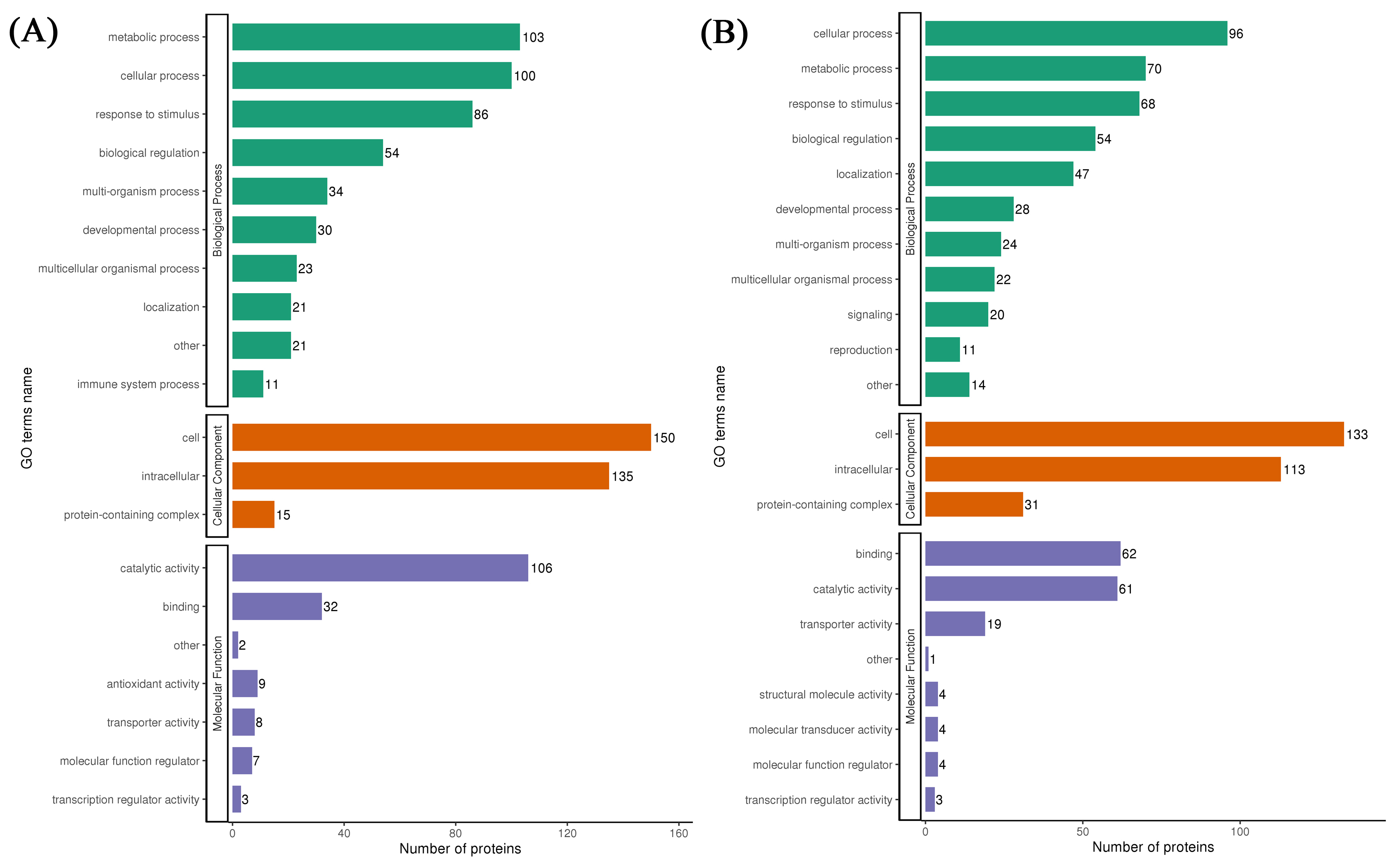

Supplement: Supplementary file 1 [file ijms-26-00634-s001.zip › Supplementary Figure S1.jpg]

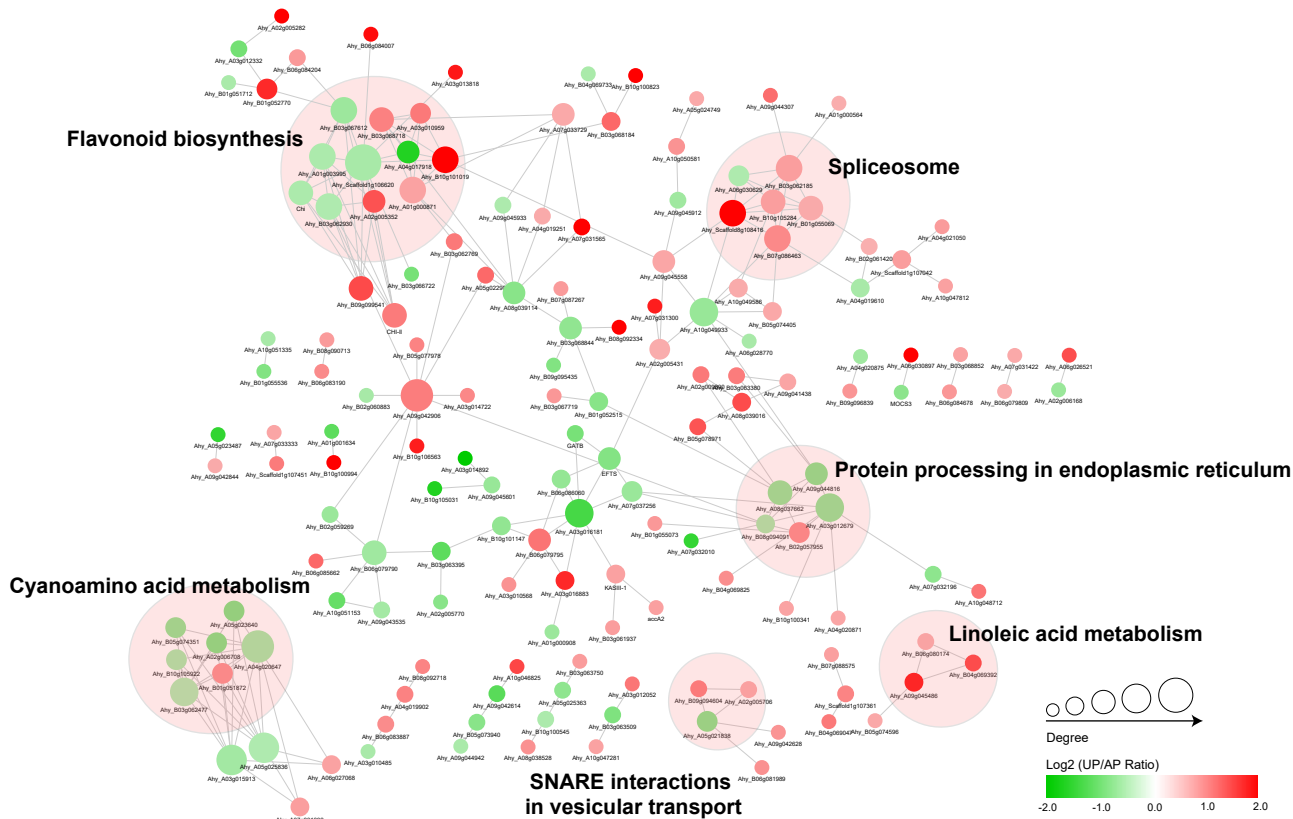

Supplement: Supplementary file 1 [file ijms-26-00634-s001.zip › Supplementary Figure S2.pdf]

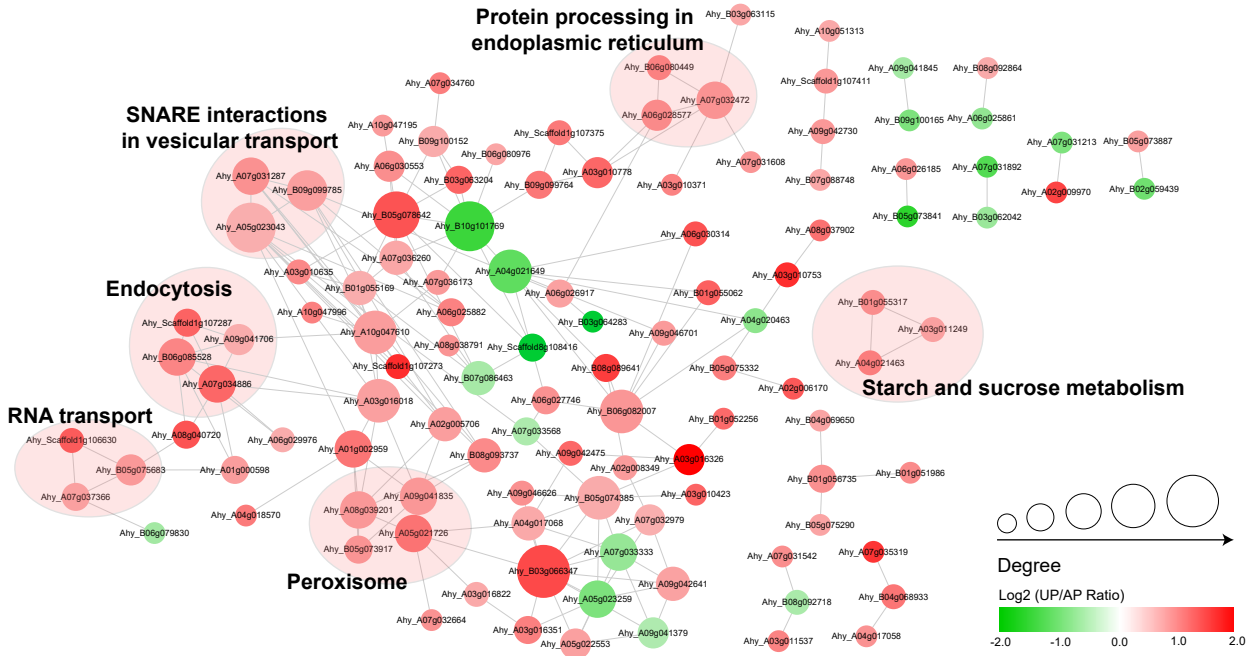

Supplement: Supplementary file 1 [file ijms-26-00634-s001.zip › Supplementary Figure S3.pdf]
